# Effects of sex and sex-related facial traits on trust and trustworthiness: An experimental study

**DOI:** 10.3389/fpsyg.2022.925601

**Published:** 2023-01-05

**Authors:** Victoria V. Rostovtseva, Marina L. Butovskaya, Anna A. Mezentseva, Franz J. Weissing

**Affiliations:** ^1^Groningen Institute for Evolutionary Life Sciences, University of Groningen, Groningen, Netherlands; ^2^Institute of Ethnology and Anthropology, Russian Academy of Sciences, Moscow, Russia

**Keywords:** trust, trustworthiness, trust game, sex differences, facial femininity, facial masculinity, sexual dimorphism, geometric morphometrics

## Abstract

The ability to trust others, including strangers, is a prerequisite for human cooperation. Economically it is not rational to trust strangers, as trust can be easily exploited. Still, generally, the level of trust toward strangers is relatively high. Trust is closely related to trustworthiness: when trusting others, one expects them to reciprocate. Some individuals elicit more trust than others. Apparently, humans use subtle cues for judging the trustworthiness of their interaction partners. Here, we report on an experiment that investigates trust and trustworthiness in a population of 176 mainly Dutch students. The aims of our study were: (1) to investigate how the sex of interaction partners and their facial appearance (femininity/masculinity) affect the degree of trust and trustworthiness, compared to fully anonymous conditions; (2) to test whether individuals who elicit trust in their interaction partners are trustworthy themselves. Each subject of our experiment played five one-shot Trust Games: one with an anonymous interaction partner, and four “personalized” games after seeing a 20 s silent video of their interaction partner (twice same-sex, and twice opposite-sex). The degree of facial sexual dimorphism was investigated with geometric morphometrics based on full-face photographs. Our results revealed that, despite the already high level of trust in the anonymous setting, the personalization of interactions had a clear effect on behavior. Females elicited more trust in partners of both sexes. Interestingly, females with more feminine faces elicited less trust in both male and female partners, while males with more masculine facial shape were more trusted by females, but less trusted by males. Neither sex nor facial femininity/masculinity predicted trustworthiness. Our results demonstrate that (1) sex and sex-related facial traits of interaction partners have a clear effect on eliciting trust in strangers. However, (2) these cues are not reliable predictors of actual trustworthiness.

## Introduction

A large part of Darwin’s (1871) *The Descent of Man* is devoted to the question whether and how humans differ from “lower animals” regarding their “social instincts” and their “moral sense,” and how and when these differences might have arisen in human evolution. The extensive cooperation between unrelated individuals, and even complete strangers who are not likely to meet again, is a striking feature of human societies, which distinguishes humans from all other organisms (Melis and Semmann, 2010). The ability to trust other individuals, including strangers, is perhaps the most important prerequisite for such large-scale cooperation. Trying to understand the nature of human trust and trustworthiness is therefore a research goal in the Darwinian tradition.

Trust as a phenomenon is being studied in various disciplines, including the social sciences, psychology, and economics (Bateson, 1988; Hardin, 2002; Uslaner, 2018; Kumar et al., 2020; Liu and Chen, 2022). The definitions of trust vary from field to field. Generally defined as “the intention to accept vulnerability based on positive expectations of the intentions or behavior of another” (Rousseau et al., 1998, p. 395), trust may also refer to such aspects as risk-taking (Luhmann, 1988), compatible interests of trustor and trustee (Hardin, 2002), and is even defined by some authors as “a bias in the processing of imperfect information about the partner’s intentions” (Yamagishi and Yamagishi, 1994, p. 136). Trust may also be manifested in sharing private information with a person, without any direct return expectations.

Here, we adopt a commonly used definition in the behavioral sciences and refer to trust as the willingness to put oneself in a vulnerable position with expectation of mutual benefits. Trust is closely related to trustworthiness: when trusting others, one expects them to reciprocate (be trustworthy; Hardin, 2002). However, trust does not imply any mechanisms of control of reciprocation, and thus can be easily exploited. This is the main reason why trusting strangers in short-term interactions is considered irrational from an economic perspective (Mas-Colell et al., 1995; Kacelnik, 2006).

Although trust is frequently manifested in human everyday life (Weiss et al., 2020), the evolutionary emergence and stability of trust is not well understood: as trust can be exploited, it should easily break down. Results of experimental studies based on the Trust Game (Kreps, 1990; Berg et al., 1995) show that humans are generally predisposed to trust other individuals (Berg et al., 1995; Ostrom and Walker, 2003; Johnson and Mislin, 2011; Wilson and Eckel, 2011; Wilson, 2018; Gómez-Miñambres et al., 2021), and that repeated interactions with the same partner are able to sustain and even reinforce trust (Anderhub et al., 2002; Bornhorst et al., 2004; Cochard et al., 2004). Interestingly, in one-shot interactions, under “all-or-nothing” condition, the fact of being trusted does not systematically induce reciprocation (Kiyonari et al., 2006), whereas in continuous versions of the game, with the option to vary the trust investment, insufficient demonstration of trust reduces reciprocal trustworthiness (Gómez-Miñambres et al., 2021).

Despite the abundance of empirical evidence that humans are generally prone to trust others, recent studies also suggest that there are considerable differences in the level of between-individual trust and trustworthiness across cultures (Thanetsunthorn and Wuthisatian, 2019; Kwantes and Kuo, 2021). And even within the same culture human trust is not indiscriminate. Studies demonstrate that social proximity has a strong effect on trust and trustworthiness (relatives, friends, and in-group members are generally trusted more than unfamiliar individuals; Dinesen et al., 2020; Porto and Pilati, 2021). The establishment of a reputation of trustworthiness in longer-term interactions or in a community is an efficient way to grow trust, but this requires the availability of reliable information about the long-term behavioral tendencies of others. Entering new social environments and interacting with strangers (which is especially common under modern urban conditions of big cities), most people do not trust indiscriminately: under such conditions, they use all kinds of information that may indicate the trustworthiness of their interaction partners. Several studies have shown that some individuals elicit more trust than others, even if the trusting person has never interacted with these individuals before (Wilson and Eckel, 2006; Rezlescu et al., 2012; Brustkern et al., 2021; Rostovtseva et al., 2022a). Apparently, humans use subtle cues to judge whether an interaction partner is trustworthy or not. A number of studies revealed that the shape of a neutral face contributes to perceived trustworthiness (Todorov et al., 2008; Stirrat and Perrett, 2010; Rezlescu et al., 2012; Kleisner et al., 2013; see also the meta-analysis of Siddique et al., 2022 and references therein). Most studies converge to the point that trustworthy-looking faces have elevated brow ridges, prominent cheekbones, lower upper facial width-to-height ratio, and wider chin. Besides this, perceived trustworthiness was reported to be positively related to facial self-resemblance (DeBruine, 2002; Farmer et al., 2014) and facial typicality (Sofer et al., 2015). Results on the association between facial attractiveness and perceived trustworthiness are contradictive (Wilson and Eckel, 2006; Sofer et al., 2015).

Here, we report on an experiment that sheds some light on the determinants of trust between strangers. The subjects in our experiment played a one-shot Trust Game (Kreps, 1990; Berg et al., 1995; Jaeger et al., 2022), which tests both their degree of trust and trustworthiness, with several other subjects. We compare the behavior of individuals in “anonymous” and “personalized” interactions. Under “anonymous” condition, they did not receive any information on their interaction partner; while under “personalized” condition they were shown a brief silent video of their partner, whom they never met before. To allude to the second half of Darwin’s (1871) book (*Selection in Relation to Sex*), we were particularly interested in sex differences regarding trust and trustworthiness. According to numerous studies, there is a persistent stereotype of women being perceived as more trustworthy than men in various aspects, such as honesty, telling the truth, and reciprocation (see the review by Schniter and Shields, 2020). Quite a number of studies examine sex differences in trust and trustworthiness under anonymous conditions of the “investment” variant of the Trust Game. Most of them indicate that without information about the sex of a partner, males are more predisposed to trust strangers than females, but there are no sex differences in trustworthiness (see the meta-analysis by Van den Akker et al., 2020). Our study was designed to address the following questions: (a) To what extent does trust and trustworthiness occur under anonymized conditions? (b) To what extent does trust and trustworthiness of individuals differ in relation to own sex and the sex of one’s interaction partner? (c) What happens if these conditions are personalized: does seeing a partner affect the behavior? (d) Are there particular facial cues that elicit trust and/or trustworthiness? and (e) Are individuals who tend to elicit trust in their interaction partners actually more trustworthy?

For the assessment of particular facial features that may contribute to eliciting trust and/or trustworthiness, we focused on facial femininity and masculinity. The literature provides some fragmental evidence that discrete facial traits hypothetically associated with sex-specific facial appearance may predict perceived and actual trustworthiness (Stirrat and Perrett, 2010; Sanchez-Pages et al., 2014). To our knowledge, the present study is the first to test whether and how facial femininity/masculinity, measured directly from the facial morphology of the studied subjects by means of geometric morphometrics, may contribute to human trust and trustworthiness.

## Materials and methods

### Overview of the experiment

The experiment we will report on is part of a larger study that was conducted in 2017 at the University of Groningen in Netherlands (Rostovtseva et al., in preparation). The participants of the study had to show up on two different experimental days. On the first day, all subjects completed general demographic questionnaires providing information on their age, ethnicity, sex (male or female), and sexual orientation (heterosexual, homosexual, or bisexual). Individual photographs and videos were taken on the first day of the experiment as well. On the second day, the participants had to take decisions in ten situations that correspond to well-known games from game theory (e.g., Dictator Game, Prisoner’s Dilemma Game, Trust Game, Stag Hunt Game, and Coordination Game). Here, we focus on the Trust Game that investigates trust and trustworthiness (see below). Each participant played five editions of the Trust Game against five different interaction partners. The first edition took place in an anonymous setting (without any information about the interaction partner); the other four took place under ‘personalized’ conditions (after having seen a 20 s silent video of the interaction partner). The interaction partners in the first two personalized editions were of the same sex, while they were of the opposite sex in the other two personalized editions. In each edition of the Trust Game, the subjects had to take a decision in two different roles: the role of a trustor and the role of a trustee (see below).

### The Trust Game

The Trust Game (TG) is a one-shot two-player game, where one of the players is in the role of trustor, while the other is in the role of trustee. Many variants of the TG have been studied in the literature (Kreps, 1990; Berg et al., 1995; Breuer et al., 2016; Andreozzi et al., 2020); here, we applied the variant of the TG where the trustor makes a binary choice, while the trustee has a range of options (Jaeger et al., 2022). In our study, the trustor was endowed with a fixed budget (50 points) and was asked to make the binary choice of either keeping the endowment or entrusting it to the other player. In the latter case, the endowment was tripled (to 150 points). Subsequently, the second player (the trustee) was endowed with these 150 points and had to decide how much to return to the trustor (options were: 100, 75, 50, or 0 points) and how much to keep for themselves (50, 75, 100, or 150 points). Returning 100 points may be viewed as the most “fair” outcome, since then the profit of 100 points is equally shared between the trustor and the trustee. However, the decision on how much to return was entirely at the discretion of the trustee, who could not be punished in any way for “unfair” behavior.

In our experiment, the Trust Game was embedded in a real-life story. The trustor was asked to imagine to be a farmer with some goods for sale (worth 50 points on the local village market), who meets a stranger on their way to the city market. The stranger offers to take the goods to the city market, expecting to sell them there for thrice the local value. The farmer then had to decide whether to accept or to decline the offer, knowing that it is solely the stranger’s decision whether to return any money (and how much). Conversely, the trustee was asked to imagine to be a seller on the city market, who was entrusted goods that could be sold for thrice the local price in the city. The decision then was whether to return any money to the farmer, and if so, how much (see Supplementary Materials for the instructions we gave to the participants).

To prevent learning effects, the participants did not get any feedback on the outcome of the interactions in the Trust Game. In particular, they did not receive any “immediate” payoffs but rather a cumulative payoff at the end of the whole experiment. The games were not real-time interactive. Instead, we first recorded the decisions of all participants in the various decision situations. Only at the end of the experiment, payoffs were assigned to these decisions, by matching each decision of a participant with a decision of the participant’s partner. In the anonymous setting, the partner was chosen at random, while in the personalized setting the partner was the person shown on the video. The partner’s decision was a decision that the partner had made in the anonymous setting, as we assumed that these decisions best reflect the behavioral predispositions of the partner. Subsequently, the payoffs were calculated according to the game rules and summed up with the payoffs from all other experimental games. The participants did not receive any information about the exchange rate between the currency used in the experimental games (points) and their final payoff in Euros; they were only informed about their overall payoffs (in Euros) at the end of the experiment. At the stage of recruitment, the subjects were told that the average *per capita* payoff for the whole experiment would be around 40 €, but that the actual payoff would strongly depend on individual performance. Therefore, participants were highly motivated in optimizing their decisions.

### Experimental procedure

Our study involved 176 subjects (87 females, 89 males). The participants were recruited by distributing flyers among the students of various BSc programs (mainly in the life sciences) of the University of Groningen and by posting a call for participants at the “Subjects Portal” of the Faculty of Behavioral and Social Sciences of the University of Groningen. All of them were 18–30 years old, with male subjects being slightly older (median = 22 years) than female (median = 21 years). Most participants (93%) were of Dutch, German, and Belgian origin (with 88% being Dutch). Five percent of male participants and 2% of female participants indicated that they were homosexual. This could have affected their behavior in same- vs. opposite-sex settings. However, the numbers were so small that it was impossible to run separate analyses on non-heterosexual participants. Excluding these participants did not affect our conclusions.

The experiment took place in computer rooms, with 15–21 same-sex participants per session. The experimental games (including the Trust Game) were implemented in the Survey Monkey Audience online form, where participants had to make their decisions by choosing options on a computer screen. Each participant was seated at a personal computer, separated from neighbors by vertical desk dividers to provide privacy. They were informed that all decisions would be treated anonymously, and others would not know their decisions at any step of the experiment. Participants were not allowed to communicate with each other during the experiment, and were asked to address all questions only to the experimenter. Each participant was asked whether they knew the partner shown in the video in person. When the answer was affirmative, the experiment continued but the case was excluded from further analysis.

### Pictures and videos

Prior to the experimental games, a silent video of each participant was recorded (20 s neutral talk to the camera), and face photographs were taken. The duration of each video was restricted to 20 s, since, according to an earlier study by other authors, 20 s is sufficient for making judgments about prosocial behavioral predispositions (Fetchenhauer et al., 2010). Videos and photographs were made under daylight condition. Each participant was seated on a fixed chair facing the window. The camera was set at the eyes’ height and at the fixed distance from an object (0.5 m for videotaping, 1.7 m for photographs). For videotaping, participants were asked to look into the camera and tell how they spent the morning. The videos were subsequently muted since voice parameters would have interfered with the effects of visual cues. Facial photographs were taken in full-face perspective with neutral facial expression, and head visually set to a natural position. Each participant was also asked to complete a general questionnaire providing information on sex, nationality, and age.

Twenty videos (10 male and 10 female) were used in the “personalised” edition of the Trust Game. These 20 videos were randomly selected from the videos of subjects of Dutch, German, and Belgian origin. According to the design of the experiment, each video was planned to be shown 40 times (20 in a same-sex, 20 in an opposite-sex setting). However, actual participation was somewhat lower; as a result, each video was displayed 31–40 times (nearly a half in the same-sex, and a half in the opposite-sex setting). Each subject played four personalized editions of the Trust Game, each with a different interaction partner, whom the subject had never met before. In general, there were 704 displays of the videos in the experiment. After the exclusion of five interactions due to personal acquaintance between the partners, 699 unique personalized interactions remained in the analysis.

### Facial morphometry

Facial shape was analyzed using geometric morphometrics (Bookstein, 1997). Photographs of individuals with beard and with considerable deviation from a natural head position were excluded from this part of the analysis, along with 13 individuals who were not of Dutch, Belgian, or German origin. Thereafter, 145 individuals (72 males, 73 females) were included in the facial shape analysis. The 20 subjects from the videos, representing interaction partners in the Trust Game, were all part of this subsample. For the analysis of associations between facial shape and behavior in the Trust Game, there remained 141 individuals, as four subjects did not show up on the second experimental day. The number of unique personalized interactions for this part was 560, as four more cases were excluded because of personal acquaintance with an interaction partner.

**Figure 1:**
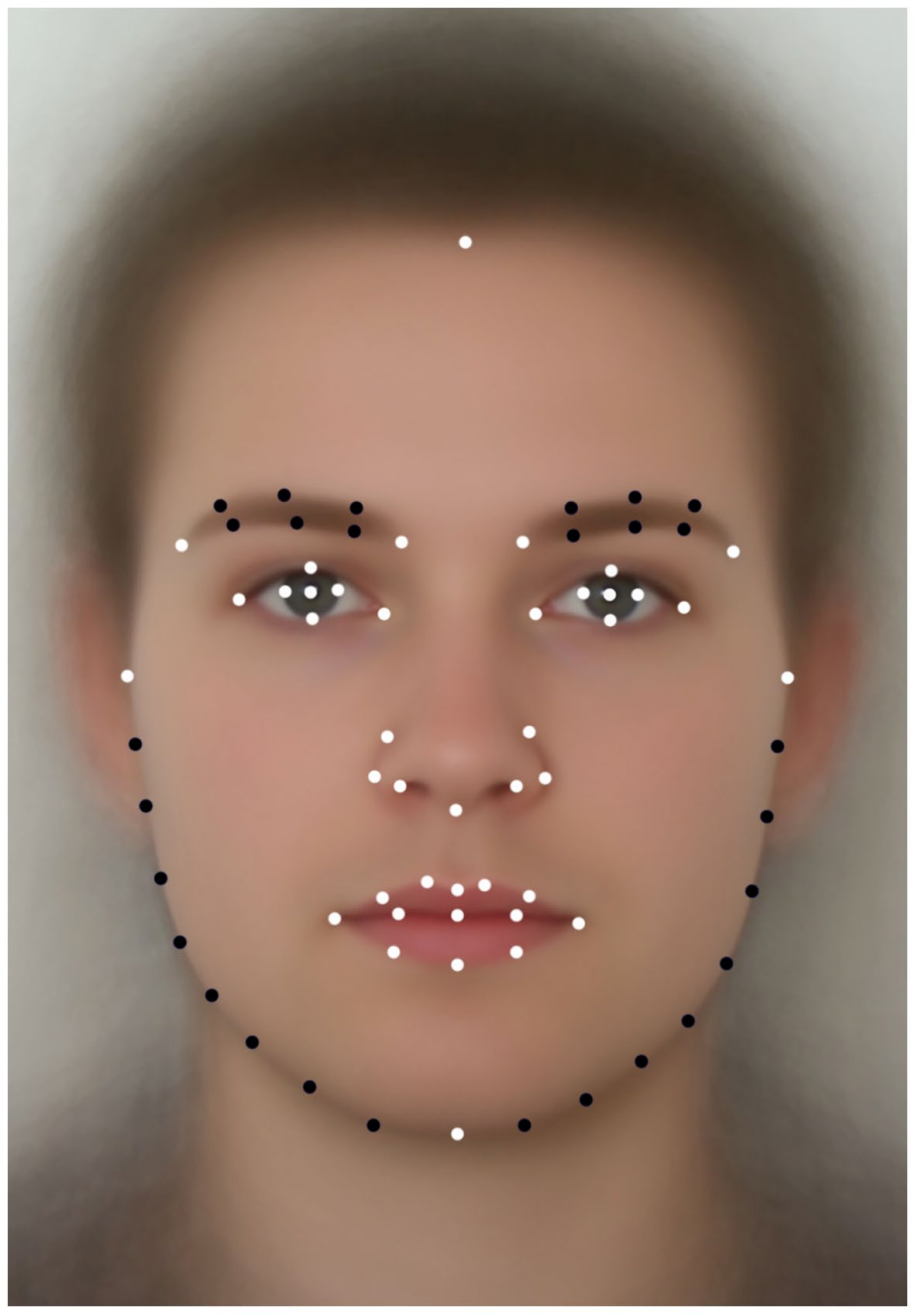
Landmarks and semi-landmarks configuration. Averaged portrait with configuration of landmarks (white) and semi-landmarks (black) is presented. For detailed description of the used anthropometric approximations see Rostovtseva et al. (2021).

Seventy landmarks and semi-landmarks were manually placed on each photograph using tpsDig2 2.17 (Rohlf, 2015). For the configuration of landmarks, we followed Windhager et al. (2011), excluding the vertex landmark that has an imprecise position when assessed from a frontal perspective. The final configuration included 36 landmarks linked to the classical anthropometric approximations to cranio-facial and soft-tissue facial shape determinants (Bunak, 1941; Alexeev and Debets, 1964; Stirrat and Perrett, 2010; Windhager et al., 2011; Tanikawa et al., 2016; Rostovtseva et al., 2021), as well as 34 semi-landmarks, which were used for covering facial outline and eyebrows shapes (Figure 1).

To test for reliability of the manual digitalization of landmarks, two independent observers placed all 70 landmarks and semi-landmarks on randomly selected photographs of 20 males and 20 females. The inter-observer agreement was assessed by means of geometric morphometrics as the ratio of among-individual variance component to the sum of among-individual and measurement error components (Zelditch et al., 2012), using the “vegan” package for R (adonis() function with Euclidian method; Oksanen et al., 2020). The inter-observer agreement was 0.96. We considered the method reliable enough to use the landmark coordinates obtained by one of the observers.

All individual facial configurations were standardized for the position, orientation, and scale by Generalized Procrustes superimposition across the general sample of 145 subjects (see Zelditch et al., 2012, for an explanation and justification of the method). Superimposition was held together with sliding semi-landmarks using minimum bending energy criterion in “geomorph” package for R (Adams et al., 2021).

Visualization of the sex differences was implemented by (i) thin-plate deformation grids, which were made in R using functions developed by Claude (2008) and adjusted by the authors according to the purpose of the present study; (ii) geometric morphometric morphs, which were created by unwarping and averaging individual photographs in tpsSuper 2.04 (Rohlf, 2015).

Individual femininity/masculinity shape scores were obtained using linear discriminant analysis for two groups (males and females) conducted on the Procrustes coordinates of the facial landmarks and semi-landmarks. The procedure was held using “MASS” package for R (Venables and Ripley, 2002).

### Statistical analysis

Statistical analysis was performed using a wide range of methods. Group differences in categorical (or binary) variables were assessed using a Chi-squared test for independence, and Fisher’s exact test (in case of small sample sizes). For estimating effects occurring under personalized condition, which implied repeated interactions with different partners, we applied generalized estimating equations (GEE) analysis with repeated measures (binary logistic and linear models with multiple predictors). Associations between two continuous variables were assessed with linear regression models, and with major axis regression using “lmodel2” package for R (Legendre, 2018). Facial shape score standardization (within each sex) was conducted using z-score transformation. A Mann–Whitney *U*-test was used to assess differences between two groups in case of asymmetrical distributions of continuously scaled variables. Most of the statistical analyses were conducted in SPSS ver. 26.0 (IBM Corp., Armonk, NY, United States). Details of the analysis are discussed in the Results section, where the statistical conclusions are presented.

Statistical analysis related to geometric morphometrics was conducted in R. Sex differences in facial shape were assessed by multivariate analysis of variance using the “vegan” package for R (adonis function with Euclidian method; Oksanen et al., 2020). Statistical significance was revealed within this function by a permutation test (with 10,000 permutations; Good, 2000).

## Results

### Behavior in the Trust Game

The participants in our experiment had to make two types of decisions: (1) in the role of trustor, they had to make the binary choice of whether or not to entrust their goods (worth 50 points on the local market) to their interaction partner; (2) in the role of trustee, who had sold the entrusted goods for 150 points elsewhere, they had to decide whether to return 100, 75, 50, or 0 points to the trustor. We consider returning 100 points a “fair” outcome, as in this case both the trustor and the trustee have a revenue of 50 points. From a purely economic perspective, the trustee should not return anything, as the interaction partners will not meet again in the future, and there is no way to punish “unfair” behavior. This, in turn, implies that, from a purely economic perspective, trustors should never entrust their goods, as these goods will be lost and no revenue will be obtained (Mas-Colell et al., 1995; Kacelnik, 2006). In contrast to this expectation, experiments based on the Trust Game typically find that human subjects have a relatively high tendency to entrust goods to their interaction partner when in the position of trustor and a relatively high tendency to return at least part of the revenue to the trustor when in the position of trustee (Berg et al., 1995; Cochard et al., 2004; Wilson and Eckel, 2011; Wilson, 2018; Andreozzi et al., 2020; see “Discussion” for more details).

Moreover, the participants had to make both types of decisions (as trustor and trustee) five times: first in an anonymous setting, where they had no information on their interaction partner; twice in a same-sex personalized setting, where they were sequentially shown two silent videos of their two different interaction partners (who were of the same sex); and twice in an opposite-sex personalized setting, where they were sequentially shown two silent videos of two other interaction partners (who were of the opposite sex).

**Figure 2:**
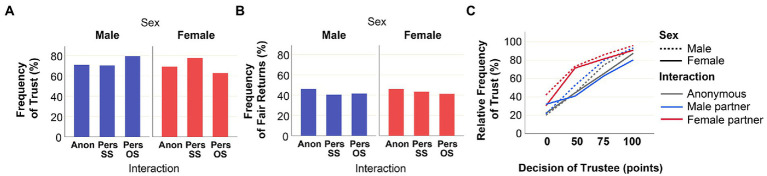
Overview of behavior in the Trust Game. **(A)** Percentage of cases where individuals in the role of trustor entrusted commodities to their interaction partner (the trustee). **(B)** Percentage of cases where individuals in the role of trustee returned a fair share of the revenue (= 100 points) to the trustor. **(C)** Association between trust (vertical axis) and trustworthiness (horizontal axis; expressed as the number of points returned to the trustee). The percentages in **(A)** and **(B)** are shown separately for males (blue) and females (red), and according to whether the decisions were taken in an anonymous setting (“Anon”), a personalized same-sex setting (“Pers SS”), and a personalized opposite-sex setting (“Pers OS”). The six curves in (C) represent the association between trust and trustworthiness for male (dashed lines) and female (solid lines) participants, dependent on whether they interacted with an anonymous partner (grey), a male partner (blue), or a female partner (red). Data from 176 subjects (89 males, 87 females) are shown, who subsequently participated in one anonymous interaction, two same-sex personalized interactions, and two opposite-sex personalized interactions. Only 699 of the 704 personalized interactions were included in the analysis (see Materials and methods).

**Figure A1:**
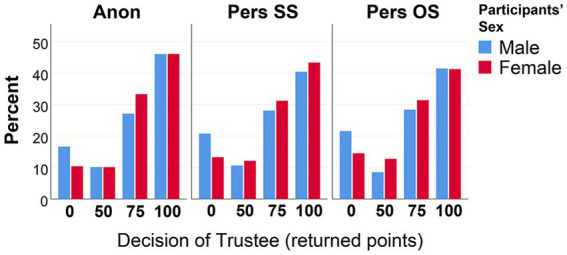
Distributions of decisions in trustworthiness part of the Trust Game. Relative frequencies (per participants’ sex) of decisions in the trustworthiness part of the Trust Game under three experimental conditions: Anon, anonymous interactions; Pers SS, personalized same-sex interactions; Pers OS, personalized opposite-sex interactions. *X*-axis represents amounts of points returned to a partner.

Figure 2 gives an overview of the behavioral decisions in our experiment. Figure 2A shows that participants of both sexes had generally a high level of trust: in the position of trustor: 70% of all subjects entrusted their commodities to their interaction partner, despite of the risk that their partner might not return anything, leaving the trustor with a loss of 50 points. Figure 2B shows that, irrespective of sex, the participants were generally trustworthy: in the position of trustee, 46% of all subjects returned the “fair share” of their revenue to the trustor (= 100 points, the maximal amount that could be returned in the experiment). As shown in Figure A1 (Statistical Appendix A), only a minority of males (17%) and females (10%) did not return anything in the role of trustee.

In cooperation experiments, one often observes a decline in cooperation tendency with the number of repetitions of a cooperation game (Andreozzi et al., 2020). In our experiment, anonymous interactions always preceded personalized interactions, and same-sex interactions always preceded opposite-sex interactions. We did not observe a systematic sequence effect on trust decisions (Figure 2A), but the frequency of fair returns, our measure of trustworthiness, slightly declined (Figure 2B).

Generally, trust and trustworthiness decisions did not differ much between anonymous and personalized conditions. In about 80% of the personalized interactions (557 out of 699) subjects made the same trust decision as in their previous anonymous interaction. Only in 142 out of 699 cases a switch occurred, either from distrust [anonymous] to trust [personalized] (*N* = 79) or from trust [anonymous] to distrust [personalized] (*N* = 63). Similarly, in about 80% of the personalized interactions (563 out of 699) subjects returned the same amount in the trustworthiness part of the game as in their previous anonymous interaction. Only in 136 out of 699 cases personalization induced a shift, either in the positive (increase in the number of points returned; *N* = 37) or the negative (decrease in the points returned; *N* = 99) direction. Within-individual consistency in the five trust- and trustworthiness-related choices can also be quantified by Fleiss’ kappa (Landis and Koch, 1977). This yields a value of *κ* = 0.451 (*p* < 0.001) for the consistency of the five trust decisions and a value of *κ* = 0.833 (*p* < 0.001) for the consistency of the five trustworthiness decisions (which were categorized on the binary scale “fair” versus “non-fair” returns to trustor, as in Figure 2B). According to the classification of Landis and Koch (1977), which is debated in the literature (Gwet, 2014), the *κ*-value for trust indicates a “moderate” level of consistency, while the *κ*-value for trustworthiness indicates “almost perfect” intra-individual consistency. These results suggest that individuals were quite consistent in their decisions, indicating that the effect of personalization on trust and especially on trustworthiness was relatively weak.

Figure 2C displays to what extent trust and trustworthiness were associated. The six curves depict the percentage of “trust” decisions in relation to the individuals’ “trustworthiness,” which is represented by the number of points returned to the trustor (0, 50, 75, 100, where 100 corresponds to a “fair” return). The curves show the associations between trust and trustworthiness separately for males and females, in each case under three experimental settings: anonymous, same-sex personalized, and opposite-sex personalized. All six curves clearly indicate a positive association between the propensity to trust and the level of trustworthiness. In other words, participants who were more predisposed to trust their interaction partners were also more trustworthy.

### Sex-related differences in trust and trustworthiness

Under anonymous conditions, male and female subjects do not seem to differ in their level of trust (Figure 2A) and trustworthiness (Figure 2B). This is confirmed by Chi-squared tests for independence that did not reveal significant sex differences in either trust (*X*^2^ = 0.069, *df* = 1, *p* = 0.792) or trustworthiness (*X*^2^ = 1.962, *df* = 3, *p* = 0.580). In contrast, a clear sex effect was observed under personalized conditions: the highest levels of trust were observed in males interacting with female partners (male opposite-sex interactions), and in females interacting with female partners (female same-sex interactions; Figure 2A). This suggests that female interaction partners elicit a higher level of trust (in both male and female subjects) than male interaction partners.

**Figure 3:**
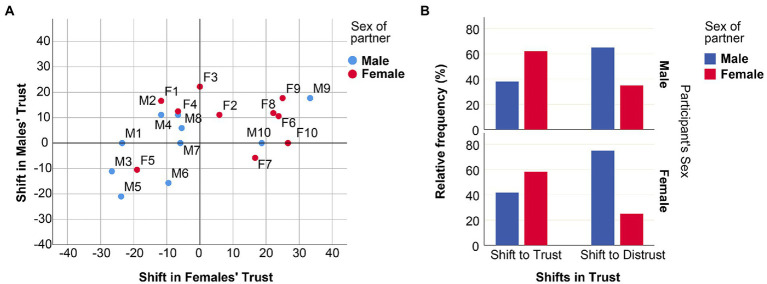
Shifts in trust decisions elicited by the 20 videos of interaction partners. **(A)** The graph depicts the average shift in trust in female (horizontal axis) and male (vertical axis) subjects elicited by each of the 20 videos of interaction partners (10 males, M1–M10, and 10 females: F1–F10). Negative scores indicate that showing the corresponding video reduced trust on average (in comparison to the trust level in the anonymous setting); positive scores indicate an increase in trust. **(B)** Relative frequency distributions of individual switches in (the binary) trust decision, in relation to the sex of the interaction partners in the Trust Game. N = 142.

Figure 3 investigates the effect of particular types of interaction partners on the behavior of male and female subjects in more detail. In the personalized setting, we made use of 20 videos (10 males: M1–M10, and 10 females: F1–F10). Each of these videos was shown to 17 ± 2 male and 17 ± 2 female subjects. The design of our study allowed us to compare, for each subpopulation of participants that had seen a given video, the frequency of “trust” decisions after having seen the video with the frequency of “trust” decisions in the anonymous setting (see Supplementary Figure 1). Figure 3A displays the shifts in trust frequency that each of the 20 videos elicited in the male and female interaction partners. A clear pattern emerges: the female videos generally led to an increase in the level of trust in both male and female participants, while the male videos led to a reduction in the trust level, especially in females.

To assess the statistical significance of the observed differences, we considered all those personalized interactions where the “trust” decision after showing the video differed from the earlier decision in the anonymous setting. Figure 3B shows how often such a switch from distrust to trust and from trust to distrust occurred for male (top graph) and female (bottom graph) participants, depending on the sex of the interaction partner (males: blue bars, females: red bars). To test for differences, we applied a binary logistic model, where a switch in the positive or negative direction was set as a response variable, where the participant’s sex, the sex of the interaction partner, and the interaction of sexes were set as independent factors. Since each decision-maker was included in the analysis four times, generalized estimating equations (GEE) with repeated measures were used. The statistical analysis (Table 1) confirms that the sex of the interaction partner is a highly significant (*p* < 0.001) predictor of shifts in trust; the negative *B*-value indicates that male interaction partners induce a decrease in trust (in comparison to the anonymous setting), while female interaction partners induce an increase in trust, both in male and female participants.

**Table 1:** Sex-related shifts in trust.

Dependent variable: Shift to trust			
Predictors	*B*	Wald *X*^2^	*p*
Sex of decision-maker (male)	0.043	0.005	0.945
Sex of interaction partner (male)	−1.472	14.152	< 0.001^***^
Sex of decision-maker * Partner’s sex	0427	0.278	0.430

Results of the GEE with repeated measures, based on a binary logistic model with two predictors and their interaction. *N*[interactions] = 142. B, coefficients; Wald *X*^2^—statistics, and associated *p* value.

We followed the same general procedure to investigate whether and how trustworthiness was affected by the sex of the interaction partner. Supplementary Figure 2 presents for each of the 20 videos shown the distributions of trustworthiness decisions in the personalized and the anonymous setting—for that subpopulation of subjects that was shown the video under scrutiny. Shifts in trustworthiness elicited by a certain partner video were quantified in two different ways: (a) by the average difference in the number of returned points between the personalized and the anonymous setting, and (b) by the difference in the frequency of “zero returns” between the personalized and the anonymous setting—where in the two methods, the average and the frequency refer to that subset of participants that was shown a given video. Again, switches in behavior were calculated separately for male and female subjects.

**Figure 4:**
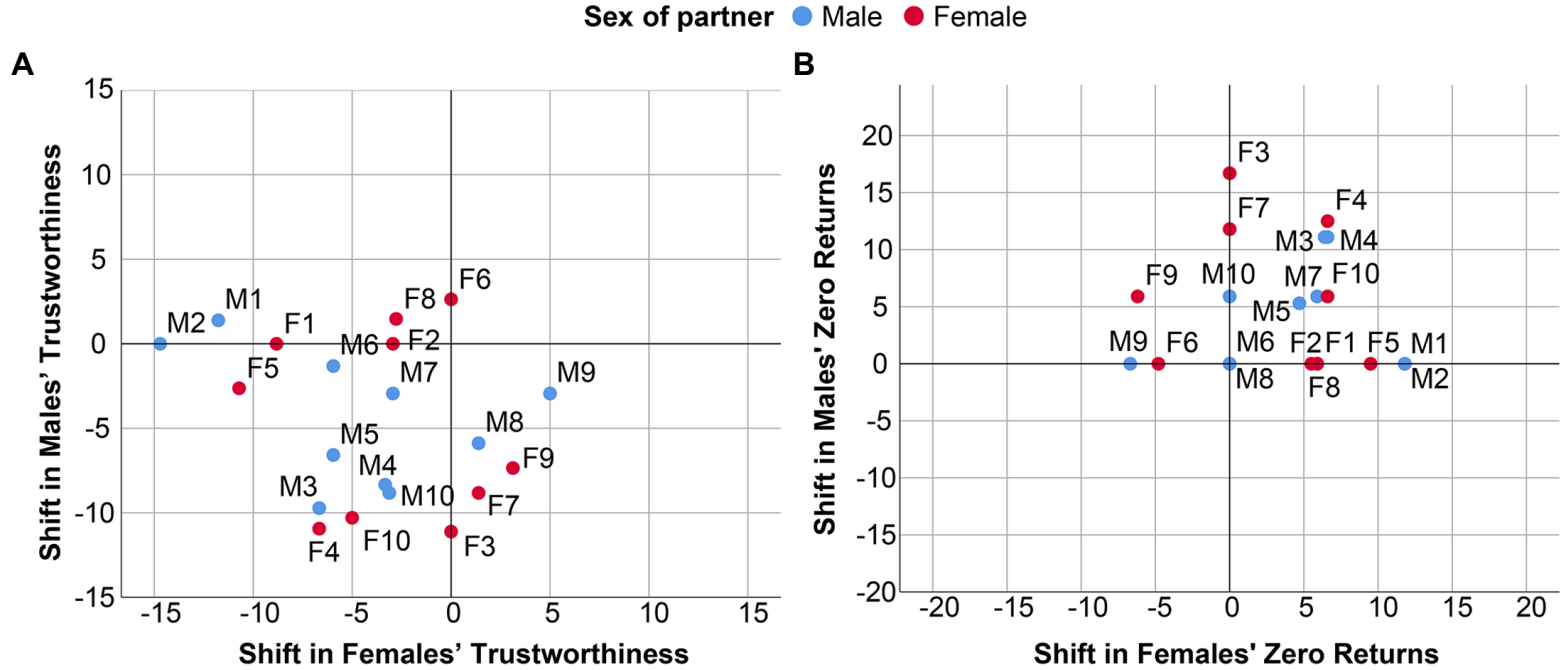
Shifts in trustworthiness elicited by the 20 videos of interaction partners. The graphs depict the average shift in trustworthiness in female (horizontal axis) and male (vertical axis) subjects elicited by each of the 20 videos of interaction partners (10 males: M1-M10 and 10 females: F1-F10). The two panels correspond to two different ways to quantify a shift in trustworthiness. **(A)** Average difference in returns in the personalized and the anonymous setting; **(B)** difference in the frequency of zero returns in the personalized and the anonymous setting. Notice that a positive value in **(B)** indicates a negative effect on trustworthiness.

Figure 4 displays for both measures the shift in trustworthiness induced by each of the 20 videos (males: M1–M10, females: F1–F10). Both panels confirm our previous observation (Figure 2B) that the level of trustworthiness was generally lower in the personalized setting than in the anonymous setting. In fact, not a single video enhanced trustworthiness (as measured by the number of points returned to the trustor; Figure 4A) in both sexes, and only 3 videos enhanced trustworthiness (as measured by the percentage of zero returns; Figure 4B) in at least one sex (females). The negative shift in trustworthiness occurred both in male and female participants, and was independent of the sex of the interaction partner. For detailed statistics see Table A1 in the Statistical Appendix A.

**Figure 5:**
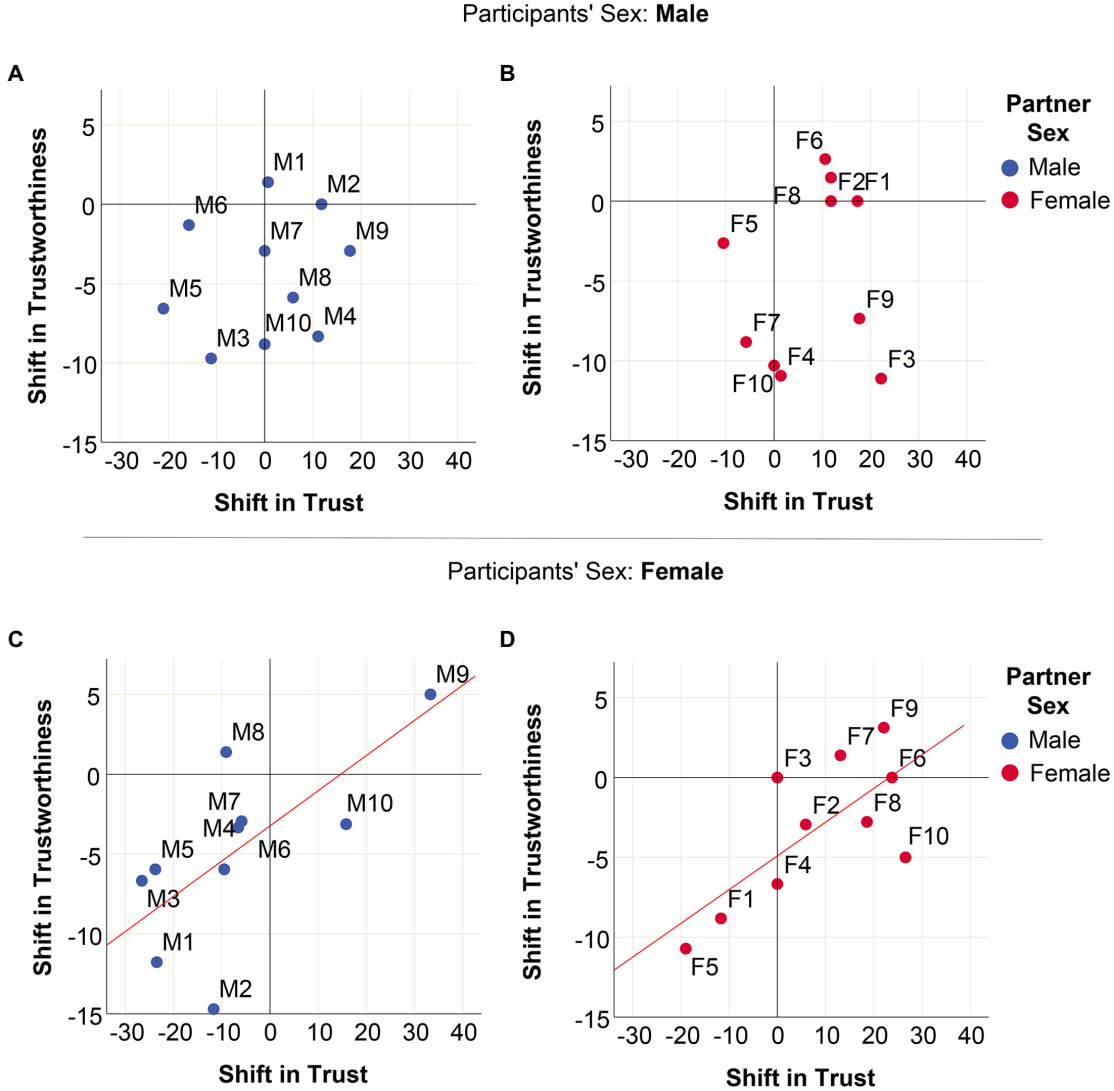
Associations between trust and trustworthiness as elicited by 10 male and 10 female videos. Major axis regression analysis of the association between shifts in trust (*x*-axis) and shifts in trustworthiness (*y*-axis) elicited by 20 videos (males: M1–M10, 10 females: F1–F10). **(A)** Effect of male videos on male subjects: *R*^2^ = 0.038, Beta = 0.066, *p* = 0.306; **(B)** effect of female videos on male subjects: *R*^2^ = 0.028, Beta = −0.115, *p* = 0.330; **(C)** effect of male videos on female subjects: *R*^2^ = 0.449, Beta = 0.217, *p* = 0.014; **(D)** effect of female videos on female subjects: *R*^2^ = 0.499, Beta = 0.214, *p* = 0.013. Major axis regression lines are presented only for significant associations.

Figure 5 depicts the associations between the shift in trust and the shift in trustworthiness that was elicited by each of the 20 videos (see the figure caption for detailed statistics). The top panels (Figures 5A,B) suggest that in male participants, there is no association between the “trust response” and the “trustworthiness response” that is elicited by male and female videos. This means that interaction partners who elicited an increase in trust in male subjects did not necessarily elicit an increase in trustworthiness in the same subjects. The outcome was very different for female participants: there was a strong and significant positive association between the trust response and the trustworthiness response that is elicited by male and female interaction partners (Figures 5C,D). Those partners from the videos who induced positive shifts in female trust also received more points from them in the trustworthiness part of the Trust Game.

### Effect of sex-related facial traits on trust and trustworthiness

**Figure 6:**
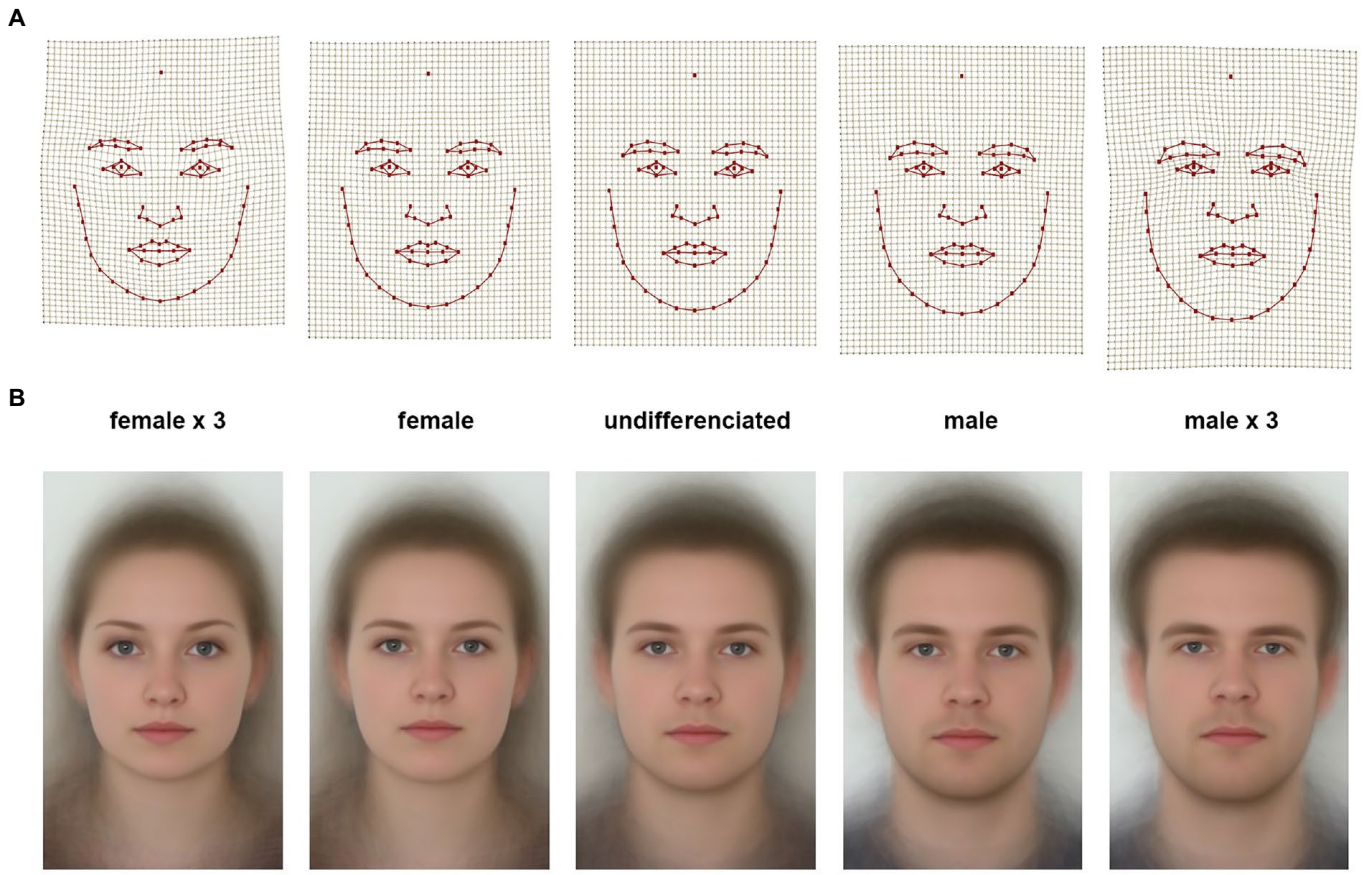
Sex differences in facial shape. **(A)** Deformation grids, and **(B)** geometric morphometric morphs display facial change from sexually undifferentiated shape (in the center) toward the average female face (left) and average male face (right). Two outer configurations display these changes exaggerated by a factor of 3. Sex differences are statistically significant (*N* = 145, var. expl. 7%, *p* < 0.001).

One of potential factors influencing the perception of partners’ appearance is the degree of facial masculinity/femininity. To test whether facial femininity/masculinity (both in males and females) serves as a stimulus to trust, we addressed possible associations between partners’ facial femininity (based on their facial photographs) and participants’ willingness to trust those partners, and to behave trustworthily toward them. The multivariate analysis of variance performed on the facial shape Procrustes coordinates by sex revealed that facial shape differed significantly between male and female subjects. Sex explained 7% of the total variance in facial shape among the participants (*N* = 145; *p* < 0.001). Adding age of the participants as the first of the two independent variables (along with sex) revealed that age explained 1.3% of the variance in facial shape (*p* = 0.03). However, after removing this component of variance, the impact of sex remained highly significant. The percent of variance explained by sex remained practically the same (var. expl. 6.8%, *p* < 0.001). The latter suggests that variation in facial shape, which occurred due to differences in age, and variation, which occurred due to sex differences, were almost independent of each other. Figure 6 displays sexual dimorphism in the facial shape of the participants. Deformation grids (Figure 6A) and geometric morphometric morphs (Figure 6B) demonstrate facial shape change from a sexually undifferentiated face (mean shape; in the center) toward the average female shape (left) and the average male shape (right). To enhance the details, the outer configurations show these differences exaggerated by a factor of 3.

The main sex differences in facial shape occurred in the shape of the lower face and the eyes area. Women, when compared to men, had a relatively shorter and narrower lower face, while male faces were relatively longer, with wider and more robust jaws. Women also had relatively fuller lips compared to men. Female eyebrows were set more laterally and relatively higher, visible areas of the eyes were larger. Since there was only one landmark on the forehead (see Figure 1), the visualization of sex differences in the forehead area along the x-axis was not informative. One of the important components of the sex differences was degree of bilateral asymmetry. Women on average had more symmetrical faces, whereas men had a clear bilateral asymmetry with “deformation” of the left side of the face (right side of the grid).

**Figure 7:**
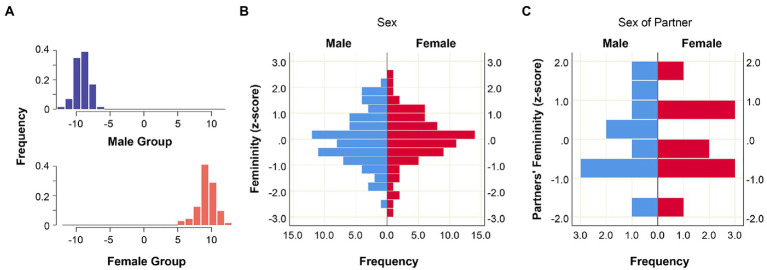
Individual facial shape scores by sex. **(A)** Facial shape scores as a result of linear discriminant analysis of Procrustes facial coordinates by sex; **(B)** distributions of femininity *z*-scores after within-sex standardization (*N* = 145); **(C)** distributions of partners’ femininity *z*-scores after within-sex standardization among partners (*N* = 20).

In order to obtain a quantitative measure of facial femininity for each participant, individual shape scores were calculated using linear discriminant analysis (LDA) for two groups (males, females) performed on the Procrustes coordinates of the facial shapes of 145 individuals. The shape scores represented the degree of facial femininity for men and women based on actual sex differences in the facial shapes in our sample. Figure 7A represents distributions of the shape scores for male and female subjects.

To use these scores as a measure of femininity of male and female faces, a within-sex z-score standardization was applied, which transformed the raw scores into standard deviations from the mean value per sex (where mean per each sex was set to zero, and ± 1 corresponded to ± 1 SD). The distributions of *z*-transformed femininity scores for males and females from the general sample are presented in Figure 7B. Our aim was to test an impact of partner’s facial femininity on shifts in trust and trustworthiness in the TG. Since the interaction partners shown at the videos were randomly chosen from the general sample, the *z*-transformed femininity scores (calculated based on facial shapes of all 145 individuals) were not distributed symmetrically between male and female partners. Although there were no statistically significant differences in femininity *z*-scores between male and female partners (Mann–Whitney *U*-test: *U* = 31.000, *p* = 0.165), there still was a small shift toward male partners having slightly higher *z*-scores than female partners (for details see Supplementary Figure 3A). Given a small sample size of interaction partners from the videos (*N* = 20) and repeated interactions (each partner’s video was shown to 17 ± 2 subjects of the same sex) this small shift still could cause biased results. For this reason, to obtain standardized femininity scores for male and female partners, the raw femininity scores were z-transform within each sex in the subsample of partners from the videos (*N* = 20). The raw femininity scores of male and female partners were normally distributed (Supplementary Figure 3B), which allowed applying z-score transformation. The distributions of the femininity *z*-scores for male and female partners are displayed in Figure 7C. Since the sample size for the facial shape analysis was reduced compared to the general sample (see “Materials and methods”), the number of shifts of personalized decisions in the Trust Game (compared to anonymous ones) was also reduced. There were 113 interactions with shifts in the trust part of the TG (65 shifts to trust, 48 shifts to distrust), and 110 shifts in the trustworthiness part (30 shifts in direction of increase of trustworthiness, 80 shifts to decrease of trustworthiness).

**Figure 8:**
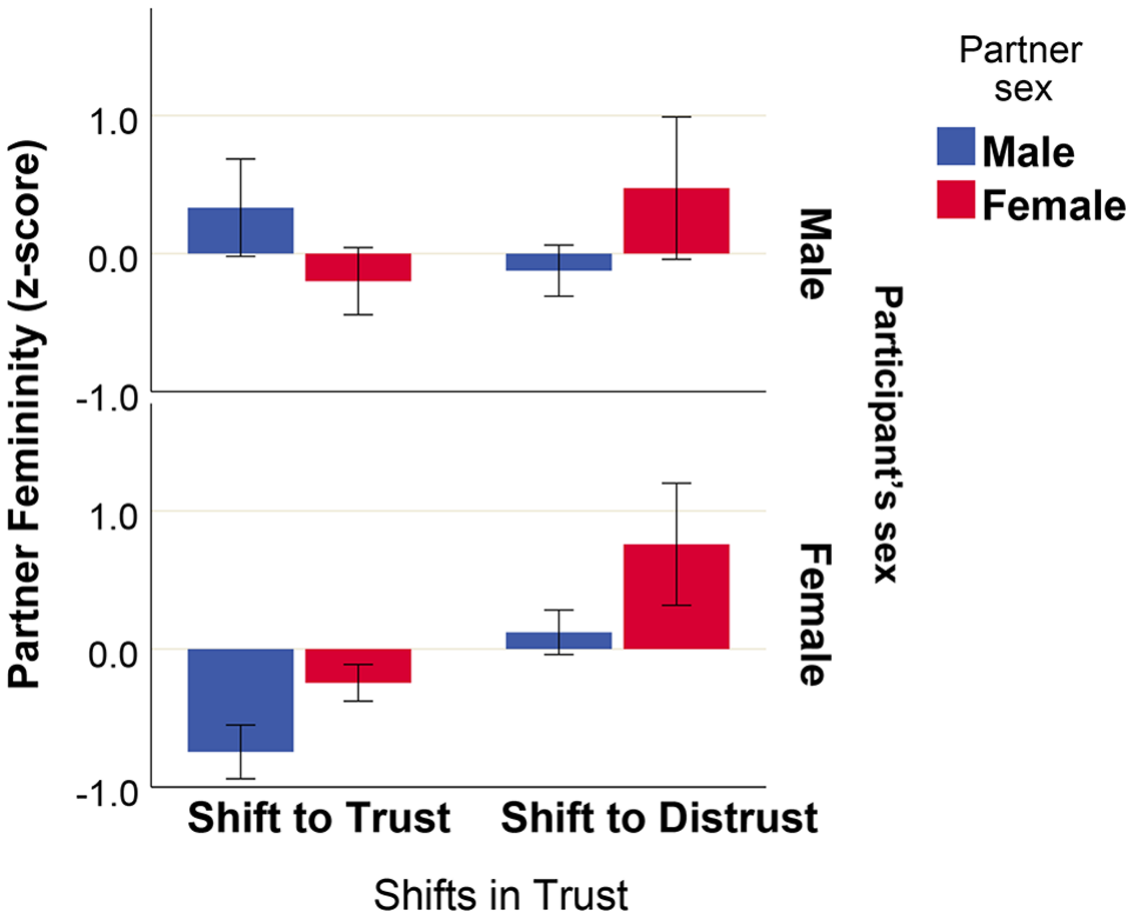
Shifts in trust, partners’ facial femininity, and partners’ sex. Associations between shifts in trust and facial femininity for interaction partners of both sexes. *N*[interactions] = 113, *N*[partners] = 20, mean values with ± 1 standard error are presented.

Our aim was to test whether a degree of facial femininity of a partner influenced shifts in trust elicited by that partner in male and female participants. Association of the partners’ facial femininity with shifts in trust, and sexes of both partners are presented in Figure 8. Although, as discussed above, females generally elicited more trust in their interaction partners (see Figure 3; Table 1), high facial femininity of female partners elicited distrust in both male and female subjects. The effects of facial femininity/masculinity of male partners were oppositely directed for male and female subjects: male participants tended to trust men with more feminine facial shapes, and female participants trusted men with more masculine faces (Figure 8).

In order to assess statistical significance of the observed effects, we used a binary logistic model, which accounted for repeated measures (GEE) based on 113 unique interactions. Shifts toward trust or distrust were set as a binary response variable, whereas sex of decision-maker, partners’ sex, and partners’ facial femininity (*z*-scores) were set as predictors (Table 2). The results confirmed that the effects of partner’s sex and partner’s facial femininity are significant predictors of shifts in trust, and that impacts of facial femininity on shifts in trust are oppositely directed for male and female subjects.

**Table 2:** Shifts in trust, degree of facial femininity, and partners’ sex.

Dependent variable: Shift to trust in personalized setting			
Predictors	*B*	Wald *X*^2^	*p*
Sex of decision-maker (male)	−0.559	0.652	0.420
**Sex of partner (male)**	**−1.473**	**8.060**	**0.005** ^**^
**Femininity of partner (*z*-score)**	**−2.836**	**11.679**	**< 0.001** ^***^
Sex of DM * Femininity of Partner (*z*-score)	**2.358**	**7.754**	**0.005** ^**^
Sex of partner * Femininity of partner (*z*-score)	0.926	1.293	0.255

Results of the GEE analysis with repeated measures are presented: a binary logistic model with multiple predictors, *N*[interactions] = 113. DM, decision-maker; *B*, coefficients; Wald *X*^2^, statistics of the Wald Chi-squared test; *p*, statistical significance (^**^p < 0.01, ^***^p < 0.001). Significant results, which remained significant after Bonferroni correction for five predictors are in bold.

**Figure A2:**
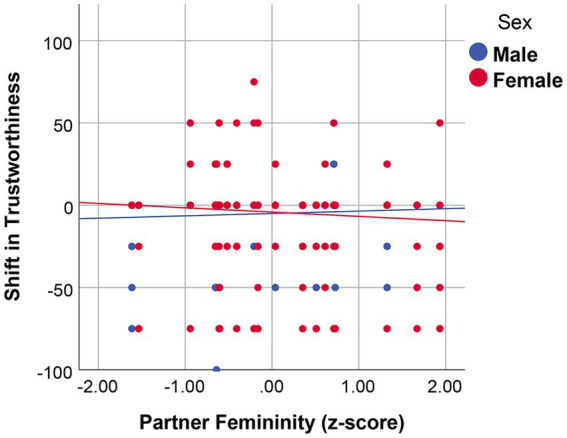
Shifts in trustworthiness, partners’ facial femininity and partners’ sex. Association between shifts in trustworthiness and facial femininity for interaction partners of both sexes (the figure depicts direction of the only significant association presented in Table A2), *N*[interactions] = 560, *N*[partners] = 20.

Next, we addressed the question of whether partner’s facial femininity/masculinity may also elicit change in trustworthiness of participants. Testing shifts in trustworthiness using the same approach as in the case of shifts in trust was not possible, since number of switches under some conditions was too small (for instance, there was only one unique interaction between two male partners with switch in direction of increase of trustworthiness). For this reason, in this part of the analysis, we measured shifts in behavior as the absolute differences in returned points between personalized and anonymous conditions per interaction. Thus, the binary variable (shift to increase, shift to decrease) turned into continuously scaled one, which was also sensitive to the magnitudes of the elicited shifts. Analysis was performed using GEE with linear model and repeated measures based on 560 unique interactions. Continuously scaled shifts in trustworthiness were set as a response variable, and sex of decision-maker, partners’ sex, and partners’ facial femininity (*z*-scores) were set as predictors. The results revealed that trustworthiness was practically not related either to facial femininity or partners’ sex. The only very weak association was found in directions of shifts in male and female participants, with feminine partners eliciting slight increase of trustworthiness in male subjects and slight decrease of trustworthiness in female subjects (Figure A2). However, this effect was very weak (*p* = 0.03), and given multiple testing could occur by chance. Detailed statistics can be found in Table A2 in the Statistical Appendix A. We conclude that partner’s sex and facial femininity did not affect subjects’ trustworthiness.

### Association of sex and sex-related facial traits with own trustworthiness

According to the obtained results, females were generally trusted more than males. At the same time, female partners with more feminine facial shape elicited more distrust in both male and female subjects, whereas male partners with more masculine facial shape were more trusted by females, and less trusted by males (Figure 8; Table 2).

Following such reactions to partners’ sex and appearance, one would expect: (i) female subjects to be more trustworthy than male subjects; (ii) females with more feminine facial shapes to be less trustworthy than females with more masculine facial appearance; and (iii) male subjects with more masculine facial shape to be more trustworthy toward female subjects, and less trustworthy toward males. In order to test whether these expectations are met, we analyzed trends in trustworthiness in association with partners’ sex and facial femininity.

It was already demonstrated that female subjects were not more trustworthy than males under anonymous condition (Figure 2B). Additional analysis also did not reveal sex differences in trustworthiness under anonymous conditions among 20 partners shown at the videos (Fisher’s exact test: *N* = 20, *p* = 0.998). Females’ trustworthiness also did not increase under personalized interactions compared to anonymous (Table 2B). These results suggest that general predisposition to trust females more than males was not justified by actual female behavior.

**Figure 9:**
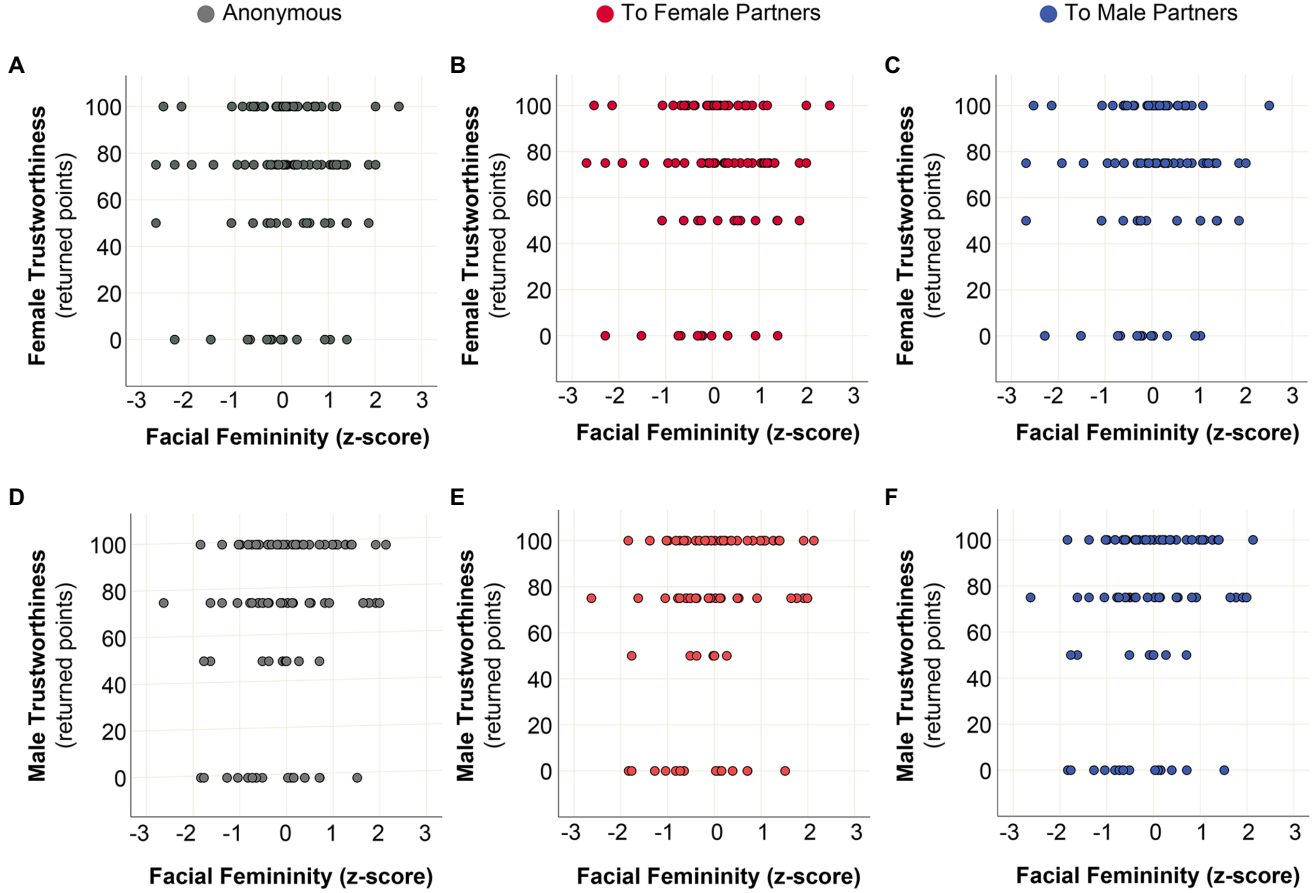
Actual trustworthiness, sex, and degree of facial femininity. **(A)** Linear regression model: response variable–female trustworthiness under anonymous condition; predictor–own facial femininity (*z*-score). *N* = 70; *B* = 2.999, *R*2 = 0.010, *p* = 0.409. **(B)** generalized estimating equations (GEE), linear model accounting for repeated measures: response variable—female trustworthiness toward female partners (personalized condition); predictor—own facial femininity (*z*-score). *N* = 139; *B* = –1.016, Wald *X*^2^ = 0.050, *p* = 0.823. **(C)** GEE, linear model accounting for repeated measures: response variable—female trustworthiness toward male partners (personalized condition); predictor—own facial femininity (*z*-score). *N* = 138; *B* = 6.312, Wald *X*^2^ = 1.171, *p* = 0.279. **(D)** Linear regression model: response variable—male trustworthiness under anonymous condition; predictor—own facial femininity (*z*-score). *N* = 71; *B* = 2.945, *R*2 = 0.008, *p* = 0.463. **(E)** GEE, linear model accounting for repeated measures: response variable—male trustworthiness toward female partners (personalized condition); predictor—own facial femininity (*z*-score). *N* = 141; *B* = –0.679, Wald *X*^2^ = 0.023, *p* = 0.880. **(F)** GEE, linear model accounting for repeated measures: response variable—male trustworthiness toward male partners (personalized condition); predictor—own facial femininity (*z*-score). *N* = 142; *B* = –0.216, Wald *X*^2^ = 0.002, *p* = 0.968.

To address more specific patterns involving both sex and femininity, we applied additional analyses. Figure 9 represents distributions of decisions in the trustworthiness part of the TG against own facial femininity under anonymous and personalized conditions for male and female subjects. Degree of facial femininity was not a significant predictor of actual trustworthiness in any of the conditions (for statistics see legend for Figure 9).

We conclude that sex and facial femininity of an interaction partner was an important factor of eliciting trust (Figure 8; Table 2). However, own sex and degree of facial femininity of participants were not reliable predictors of their actual trustworthiness (Figure 9).

## Discussion

More than two hundred studies on behavior in the anonymous Trust Game have been conducted in the last decades (games with binary-choice trust decisions: Bolle, 1998; Eckel and Wilson, 2004; Gómez-Miñambres et al., 2021; Jaeger et al., 2022; games with continuously scaled trust decisions are extensively reviewed by Ostrom and Walker, 2003; Johnson and Mislin, 2011; Van den Akker et al., 2020). Research on the effects of personalization on between-individual trust and trustworthiness became popular only recently. These personalization studies can be categorized into three lines of research. First, in quite a number of studies subjects are asked to rate neutral photographs of their interaction partners regarding various traits (e.g., trustworthiness); subsequently, these studies explore whether and how these ratings are associated with the trust and trustworthiness decisions toward these partners in the Trust Game (Bente et al., 2014; Yu et al., 2014; Li et al., 2017; Qi et al., 2018; Brustkern et al., 2021; Li et al., 2021; Shen et al., 2021). A second set of studies investigates how adding emotional facial expressions (smiles, anger displays, etc.) to a partners’ image affect trust and trustworthiness (Campellone and Kring, 2013; Tortosa et al., 2013; Alguacil et al., 2015; Kret and De Dreu, 2019; Li et al., 2021). A third line of research studies whether facial self-resemblance has a positive effect on trust and trustworthiness (DeBruine, 2002; Farmer et al., 2014). Most personalization studies are based on showing pictures of the interaction partners; only a few studies investigate trust and trustworthiness under conditions of face-to-face interactions or with dynamic partner representation (videos or dynamic avatars; Krumhuber et al., 2007; Centorrino et al., 2015; Tognetti et al., 2018; Rychlowska et al., 2019). In the present work, we made use of both, a dynamic representation of the interaction partners (videos) in the Trust Game and the analysis of full-face photographs.

The design of our study allowed us to compare personalized and anonymous interactions, and to estimate whether trust directed toward particular partners is justified by the actual trustworthiness of these partners. In addition, it enabled us to investigate how the sex of interaction partners and their facial appearance (femininity/masculinity) affect trust and trustworthiness.

Even under anonymous conditions, trust and trustworthiness were manifested at a remarkably high level in our study population: the majority of participants were generally willing to entrust goods to anonymous partners (70% of all cases), and were also trustworthy in that they frequently returned a fair share of the goods entrusted to them (46% of cases). Earlier studies by other authors revealed that in experiments trust levels tend to be higher in binary-choice variants of the trust game than in “investment games” with a continuum of choices of the trustor (Bolle, 1998; Eckel and Wilson, 2004; Gómez-Miñambres et al., 2021). At the same time, binary-scaled (“all-or-nothing”) trust may also lead to a decrease in reciprocal trustworthiness (Ostrom and Walker, 2003; Gómez-Miñambres et al., 2021). However, our results confirm earlier findings (reported for both variants of the game) that in Europe and North America, a high degree of trust and trustworthiness toward anonymous strangers is quite typical in experimental Trust Games (Berg et al., 1995; Cochard et al., 2004; Eckel and Wilson, 2004; Wilson and Eckel, 2011; Wilson, 2018; Andreozzi et al., 2020; Müller and Schwieren, 2020; Gómez-Miñambres et al., 2021). Such behavior cannot be explained by economic rationality, since reciprocation cannot be enforced by the trustor, and trust can easily be exploited by returning an unfair share or even nothing. In other words, human trust and trustworthiness are far beyond purely economic self-interest. However, the literature suggests that there are considerable population differences in the level of trust. According to the World Value Survey (World Values Survey Wave 7, 2017–2021), when subjects were asked whether they trust people whom they meet for the first time the expressed levels of trust are the highest in Denmark [75.3%], followed by Sweden [73.9%] and Netherlands [71.6%], the country where our study was conducted. The lowest levels of trust toward strangers were reported for Ecuador [7.8%], Albania [8.7%], and Peru [9.0%]. Some authors suggest that the explanation for the observed population differences may be rooted in different socio-environmental factors, which moderate trusting attitudes through social learning and everyday experiences (Kwantes and Kuo, 2021; Liu and Chen, 2022). The correspondence between the level of trust in the anonymous version of our Trust Game [70%] and the outcome of the WVS for Netherlands [71.6%] is more than a coincidence: a study across 35 countries (involving more than 23,000 subjects) revealed that the level of trust per country as measured in anonymous one-shot Trust Games aligned well with the level of trust as measured by the WVS. Interestingly, the experimentally measured trustworthiness levels were not correlated with the trust levels as measured by the WVS in those countries (Johnson and Mislin, 2012).

Under anonymous conditions, we did not find sex differences in trust or trustworthiness in our study. Earlier, van den Akker et al. (2020) conducted a meta-analysis on sex differences in trust and trustworthiness in the one-shot anonymous continuously scaled Trust Game, based on 77 articles with 174 studies in 35 countries. They concluded that men are generally more trusting than women, whereas no significant sex differences in trustworthiness are observed. As trust toward strangers is a risky behavior that may lead to higher gains, but also may lead to loss, this finding may not be unexpected, as males tend to be more risk-prone than females. In contrast to this general trend, we did not find sex differences in trust between male and female subjects under anonymous “all-or-nothing” conditions. Such inconsistency may be rooted in the differences in design (binary- vs. continuous-trust decisions) and should be studied more carefully in the future. Another explanation for this may be related to the fact that our study was conducted in Netherlands, where differences between the sexes have diminished continually in the last 50 years.

When comparing personalized with anonymous interactions, we did not observe a systematic decline or increase of trust. Generally, a high degree of consistency in individual behavior was observed across all five interactions (anonymous and personalized). There was a slight decline in trustworthiness under personalized conditions in comparison to anonymous ones. This could partly have been caused by sequence effects: the set-up of our study necessitated that anonymous interactions preceded personalized interactions, and that same-sex personalized interactions preceded opposite-sex interactions. Although this sequence may have affected our results, we tried to minimize such effects by not providing our participants with feedback on the outcomes of earlier interactions, thereby eliminating the most obvious learning effects. However, due to repetition, subjects had more time for reflection (Kahneman, 2011), which could cause a slight downward bias along the sequence of interactions.

Although there were no sex differences in trust and trustworthiness under anonymous conditions, in personalized setting, significant sex effects were revealed. In a personalized setting, females elicited an increase of trust in both male and female subjects, whereas the trustworthiness of a subject remained unaffected by the sex of the partner. This finding contrasts with conclusions drawn in the literature. One study (Scharlemann et al., 2001) found that female images elicited more trust in males, while male images elicited more trust in females. Another study (Wilson and Eckel, 2006) reports that females are more trustworthy toward males than toward females. The study investigating facial trustworthiness perception using neuroimaging (Dzhelyova et al., 2012) reports that female appearance is generally perceived as more trustworthy than male appearance. Our results support the notion that females generally elicit more trust than males, but due to the apparent plasticity of trust in different socio-cultural environments generalizations should be made with caution.

In our study, trust and trustworthiness toward the same interaction partner were strongly positively correlated in female subjects, while there was no such correlation in male subjects (Figure 5). This means that if a male participant trusted someone he did not necessarily behave trustworthily toward that partner, whereas females trusted and were trustworthy toward the same person to a similar degree. This finding suggests that male and female participants differ in behavioral strategies and/or motivations for trust.

When considering the effects of specific facial features of a partner (facial femininity/masculinity), our study revealed that, although females generally were more trusted than males, females with more feminine facial traits elicited less trust than females with less feminine faces. At the same time, males with more masculine facial traits were more trusted by female partners, but were less trusted by male partners. Although the relatively small sample of partner videos (10 males and 10 females) is a certain limitation of our study, it is reassuring that our main results are consistent with the literature. The revealed effects are a good illustration of the differential impact of male masculinization on the behavior of interaction partners of the same and the opposite sex. On the one hand, according to the literature, men with highly masculinized faces are generally perceived as less trustworthy (Stirrat and Perrett, 2010), and were reported to actually demonstrate less trustworthiness in the Trust Game (Stirrat and Perrett, 2010; Sanchez-Pages et al., 2014). On the other hand, females tend to find masculinized faces more attractive for short-term relationships (Little, 2014), which may modulate their behavior in the Trust Game (for the wrong reasons). By the same logic feminized faces should increase trust in males, while the opposite pattern was observed in our study. Indeed, a number of studies have shown that more feminine female faces are perceived as more attractive than more masculine female faces (Perrett et al., 1998; Penton-Voak et al., 2004; Rhodes, 2006). However, in one study of Hu et al. (2018), conducted in China, it was revealed that among generally attractive-looking individuals more masculine female faces were perceived as more trustworthy by both male and female subjects than less masculine faces.

Finally, we found that neither the sex nor the sex-related facial appearance of an interaction partner seems to be a reliable predictor of actual trustworthiness. This contrasts with previous studies that reported significant associations of facial features (Stirrat and Perrett, 2010; Sanchez-Pages et al., 2014) with the trustworthiness of a person in a binary-choice Trust Game. However, other studies also concluded that assigning behavioral traits to individuals with particular facial appearances (based only on visual cues) often fails to correctly predict their actual behavioral predispositions (Bonnefon et al., 2015). A recent study (based on the same variant of the Trust Game as used in our study) also showed that people are unable to detect the trustworthiness of strangers through their facial appearance (Jaeger et al., 2022). One of the possible reasons for the lack of actual recognition may be rooted in the so-called prosocial mimicry (or “camouflage”; Dawkins, 1976; Gambetta, 2005; Mokkonen and Lindstedt, 2016) used by free-riders (or cheaters) to imitate behavior and even appearance of prosocial individuals (Rostovtseva et al., 2022a,b). Although a fraction of such “cheaters” among trustworthy-looking individuals may be small, this still can introduce noise to the data. Another possible reason is that the “trustworthiness decisions” (and their interpretation) are more complicated than “trust decisions,” as the former highly depend on what one considers fair. Today it is known that the perceptual “trustworthiness stereotype” (whom to consider trustworthy? whom to trust?) is a very dynamic phenomenon, and such stereotype can be very quickly rebuilt within a single individual through social learning (Chua and Freeman, 2022).

Summarizing, our findings indicate that personalization of interactions has a clear effect on trust, but not trustworthiness. Overall, there was no positive or negative effect of personalization on trust and trustworthiness, but there was a clear effect related to sex. Our results support previous findings by other authors that females generally elicit more trust than males. However, females with more feminine facial shapes elicited less trust in both male and female partners, while males with more masculine facial shape were more trusted by females, but less trusted by males. This result indicates the differential impact of male masculinization on the behavior of interaction partners of the same and the opposite sex. Importantly, sex and sex-specific facial appearance do not seem to be reliable predictors of trustworthiness. However, one should keep in mind that our study population is ‘Western, educated, industrialized, rich, and democratic’ (WEIRD; Henrich et al., 2010) and therefore not representative of all humans.

## Data availability statement

The datasets presented in this study can be found in online repositories. The names of the repository/repositories and accession number(s) can be found at: FigShare.com, the data are available at doi: 10.6084/m9.figshare.21300924.

## Ethics statement

The study received approval by the Ethical Committee Psychology (ECP) of the University of Groningen (Research Code: 16250-0). Prior to the experiment, all subjects signed informed consents.

## Author contributions

VR, FW, and MB contributed to conception and design of the study. VR and FW conducted the experiment, analyzed the data and drafted the manuscript. VR and AM implemented geometric morphometrics. All authors contributed to the article and approved the submitted version.

## Funding

The experiment was conducted with the support of the Dr. J.L. Dobberke Foundation, and the Erasmus Mundus Action 2 programme EMA2 Aurora II (2013–2021); the data analysis and manuscript preparation were supported by the grant 18-18-00075 of the Russian Science Foundation (on behalf of VR, AM, and MB). FW acknowledges funding from the European Research Council (ERC Advanced grant no. 789240).

## Conflict of interest

The authors declare that the research was conducted in the absence of any commercial or financial relationships that could be construed as a potential conflict of interest.

## Publisher’s note

All claims expressed in this article are solely those of the authors and do not necessarily represent those of their affiliated organizations, or those of the publisher, the editors and the reviewers. Any product that may be evaluated in this article, or claim that may be made by its manufacturer, is not guaranteed or endorsed by the publisher.

## Statistical Appendix

**Table A1:** Sex-related shifts in trustworthiness.

Model	Dependent variable: Shift in points returned			
	Predictors	*B*	Wald *X*^2^	*p*
(a)	Sex of decision-maker (male)	−0.634	0.796	0.372
	Partner’s sex (male)	−0.176	0.309	0.579
	Sex of decision-maker * Partner’s sex	−0.517	0.362	0.547
(b)	**Dependent variable: Shift in frequency of zero returns**			
	Predictors	*B*	Wald *X*^2^	*p*
	Sex of decision-maker (male)	2.125	2.904	0.088
	Partner’s sex (male)	−0.231	0.249	0.617

Results of the GEE with repeated measures are presented: binary logistic model with two predictors and their interactions (for the model [b] interaction of predictors was inapplicable). (a) *N*[interactions] = 136; (b) *N*[interactions] = 54. B, coefficients; Wald *X*^2^, statistics of the Wald Chi-squared test; *p*, statistical significance.

**Table A2:** Shifts in trustworthiness, degree of facial femininity and partners’ sex.

Dependent variable: Increase of trustworthiness in personalized setting			
Predictors	*B*	Wald *X*^2^	*p*
Sex of decision-maker (male)	−0.898	0.104	0.747
Sex of partner (male)	−2.064	3.010	0.083
Femininity of decision-maker (*z*-score)	3.558	3.099	0.078
Femininity of partner (*z*-score)	−3.076	2.740	0.098
Sex of DM * Femininity of DM (*z*-score)	−0.378	0.018	0.894
Sex of DM * Femininity of partner (*z*-score)	4.354	4.734	0.030*
Sex of partner * Femininity of DM (*z*-score)	0.972	1.292	0.256
Sex of partner * Femininity of partner (*z*-score)	0.646	0.101	0.751

Results of the GEE analysis with repeated measures are presented: a linear model with multiple predictors, *N*[interactions] = 560. DM, decision-maker; B, coefficients; Wald *X*^2^, statistics of the Wald Chi-squared test; *p*, statistical significance (^*^*p* < 0.05).
