# Supplementary material for: Effects of sex and sex-related facial traits on trust and trustworthiness: An experimental study
Source: Front Psychol. 2023 Jan 5;13:925601. doi: 10.3389/fpsyg.2022.925601 (PMC9849902; doi:10.3389/fpsyg.2022.925601)
Supplement: Supplementary file 1 [file Data_Sheet_1.PDF]

Supplementary Figure 1. Frequencies of trust decisions

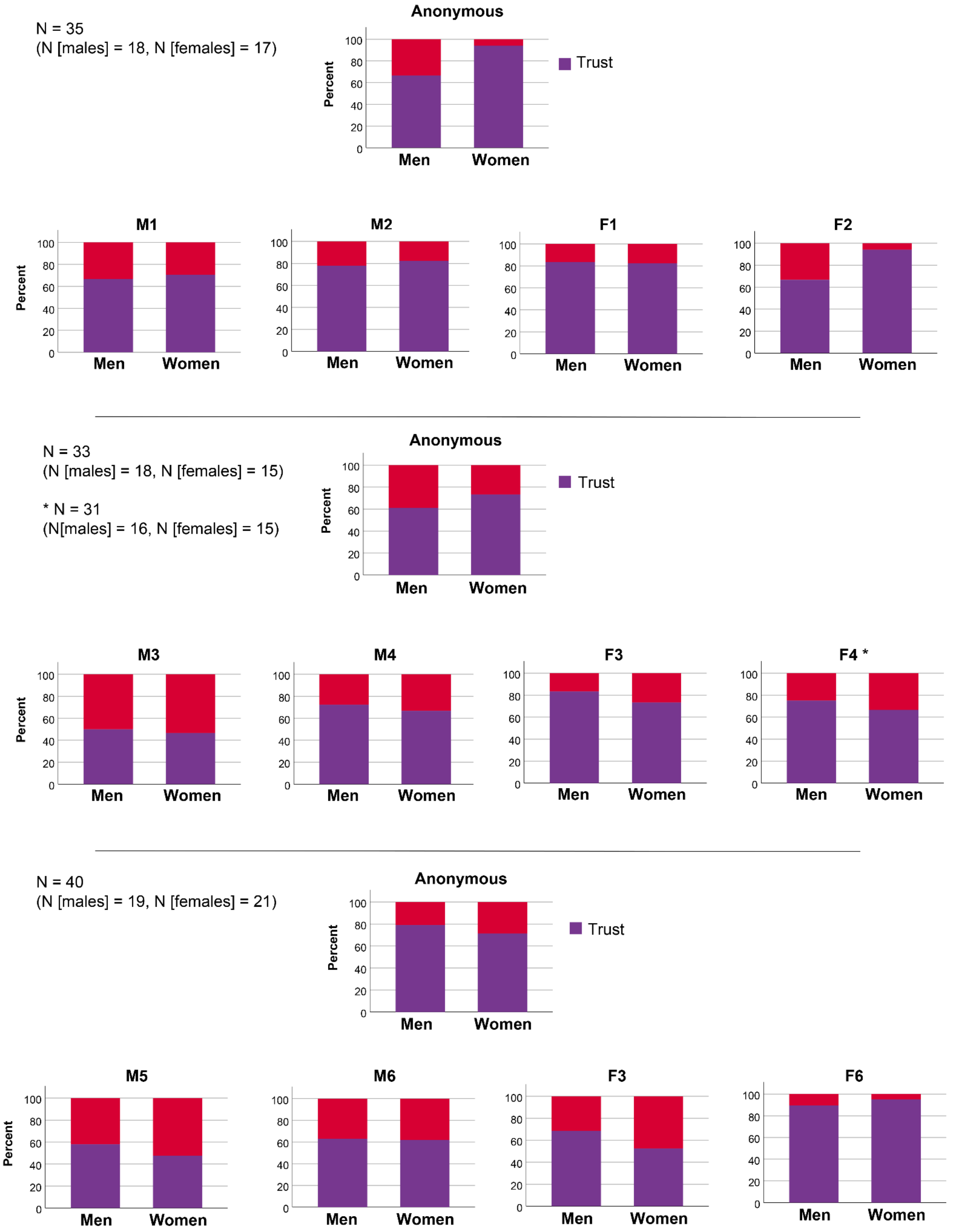

Supplementary Figure 1 (extension)

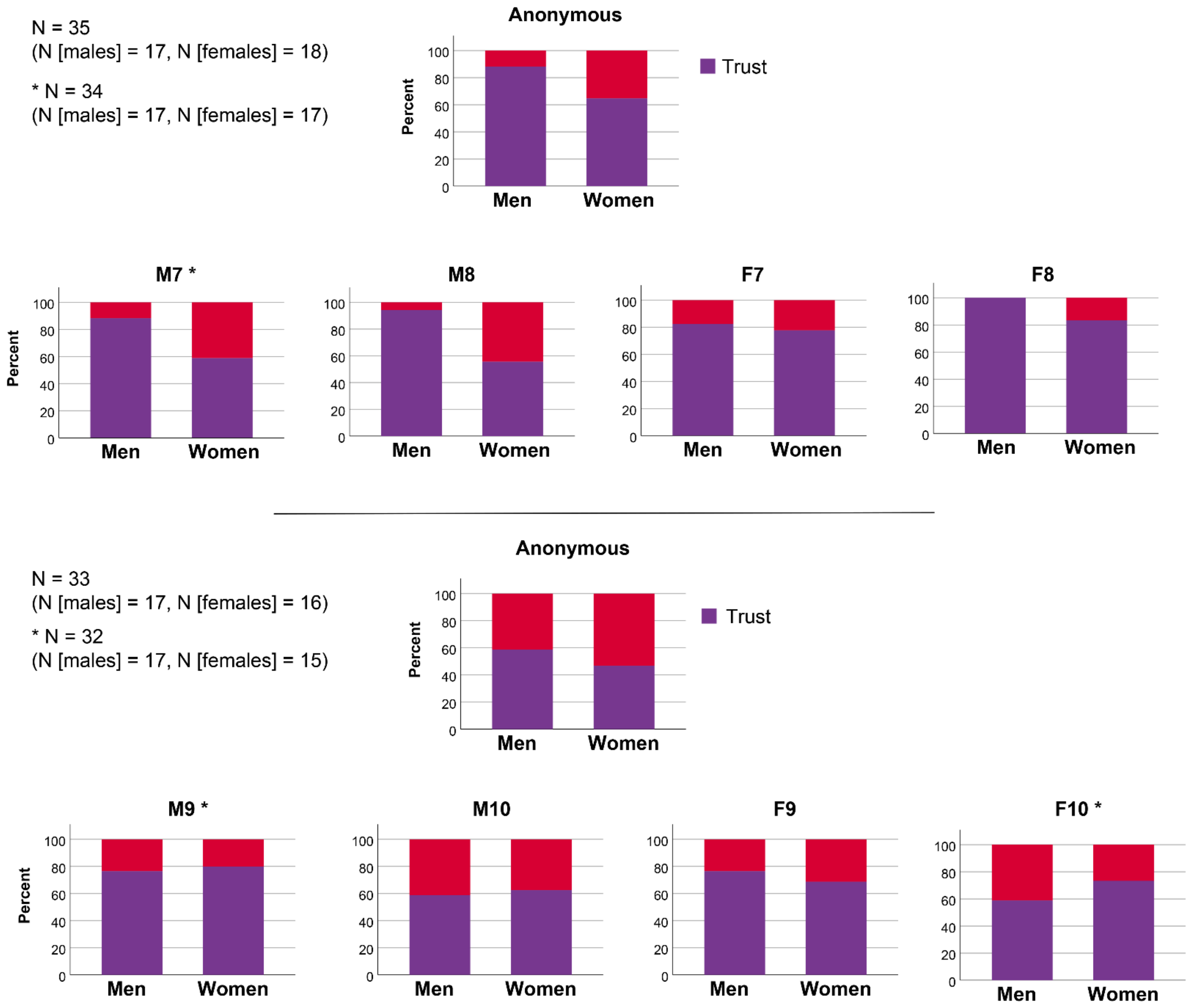

* Five personalized interactions were excluded, since those participants knew partners shown at the videos in person. Frequencies of anonymous decisions are presented for the subsamples without excluded cases. For calculations of shifts in trust elicited by each partner (video) (10 male [M1-M10] and 10 female [F1-F10] partners), a number of individuals involved in anonymous interactions per subsample strictly corresponded to a number of individuals involved in personalized interactions with a given partner.

Supplementary Figure 2. Frequencies of trustworthiness decisions

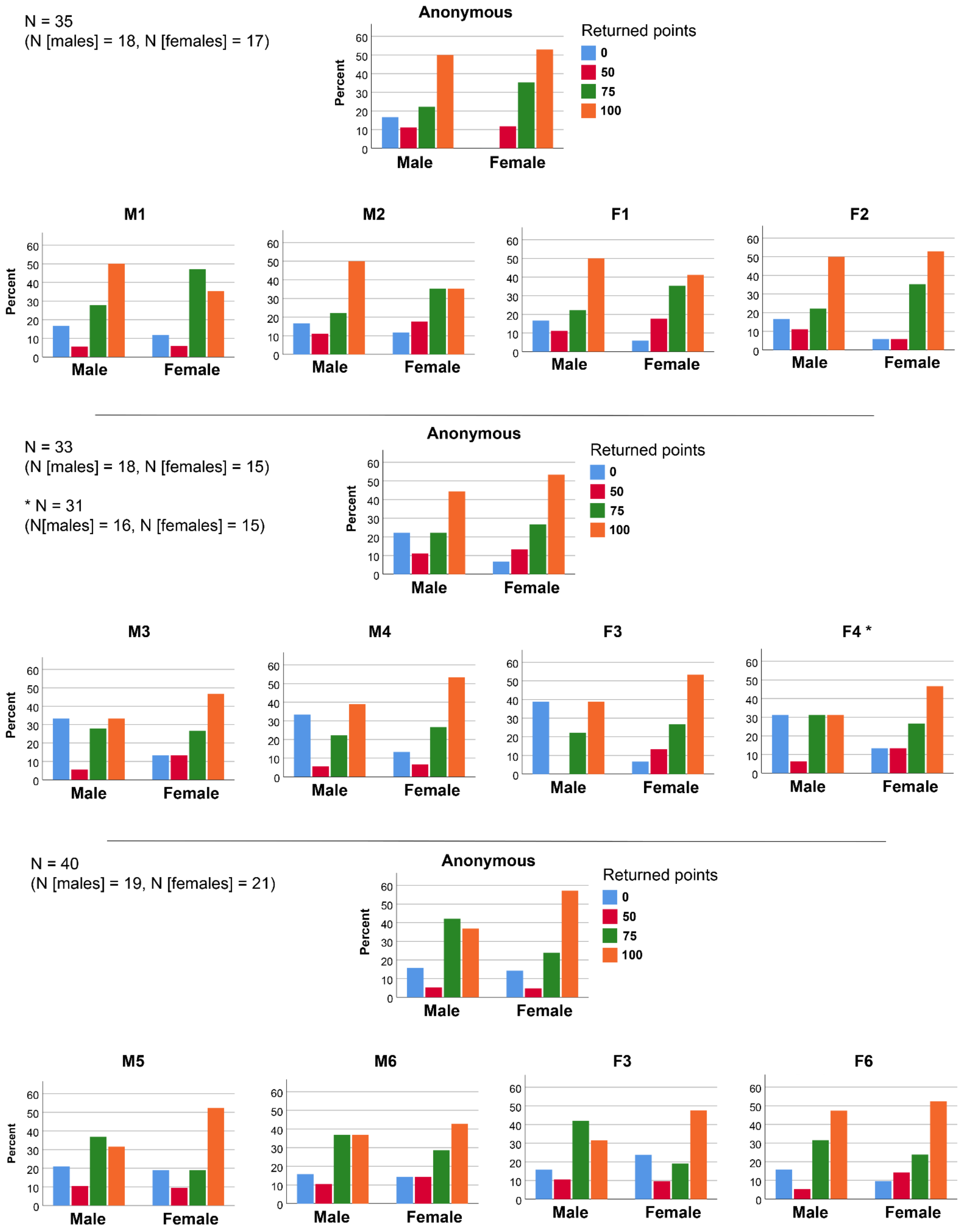

Supplementary Figure 2 (extension)

N = 35
(N [males] = 17, N [females] = 18)

* N = 34
(N [males] = 17, N [females] = 17)

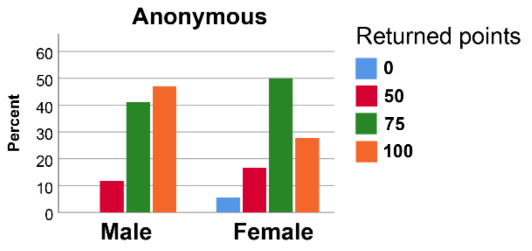

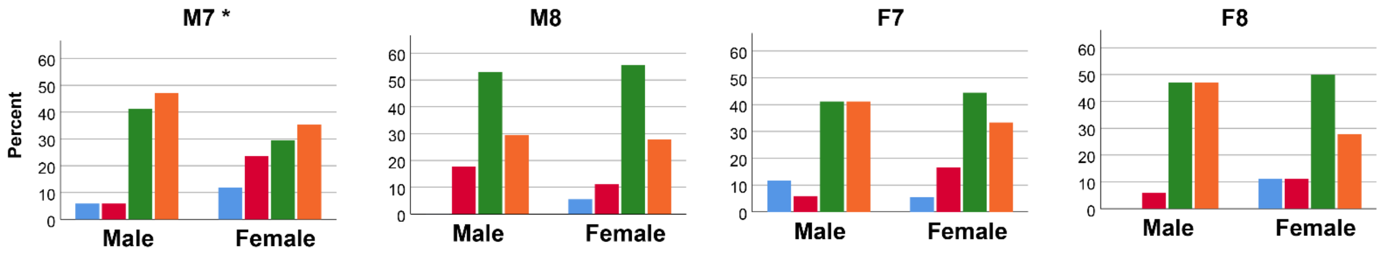

N = 33
(N [males] = 17, N [females] = 16)

* N = 32
(N [males] = 17, N [females] = 15)

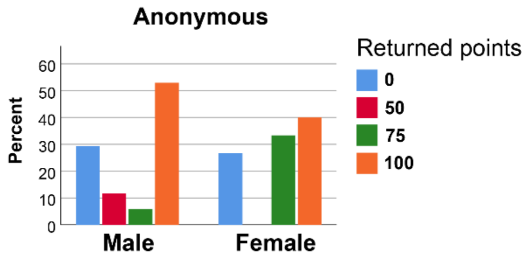

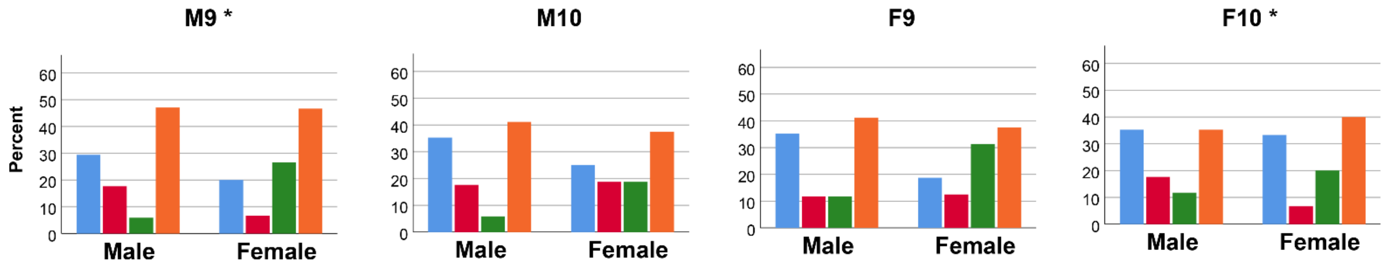

* Five personalized interactions were excluded, since those participants knew partners shown at the videos in person. Frequencies of anonymous decisions are presented for the subsamples without excluded cases. For calculations of shifts in trustworthiness elicited by each partner (video) (10 male [M1-M10] and 10 female [F1-F10] partners), a number of individuals involved in anonymous interactions per subsample strictly corresponded to a number of individuals involved in personalized interactions with a given partner.

Supplementary Figure 3. Distributions of partner femininity z-scores (a), and partner femininity scores (b)

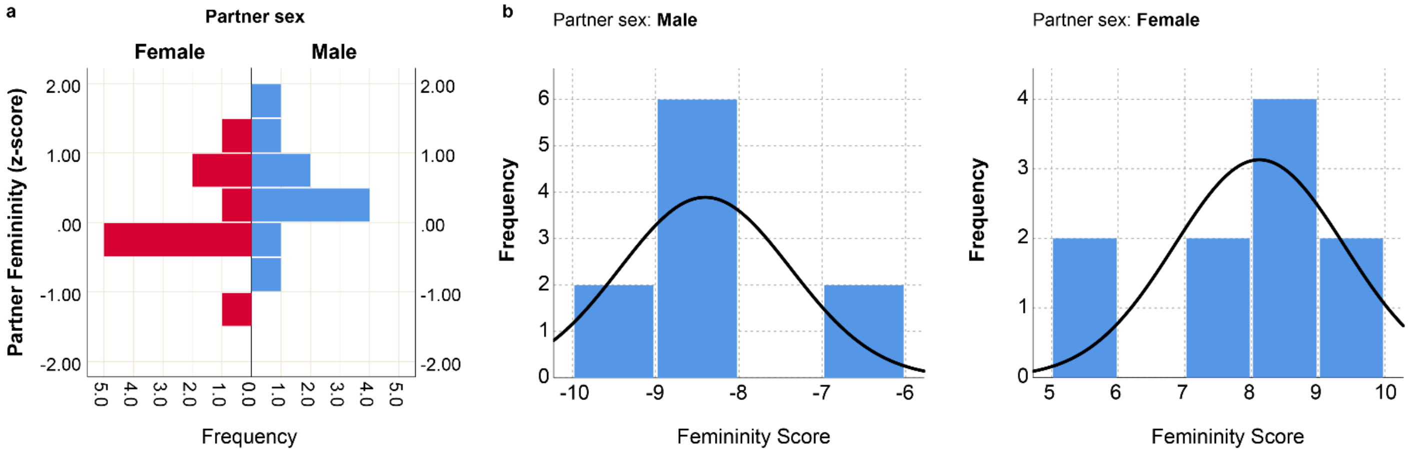

(a) z-scores were calculated based on the general sample (N=145); (b) one-sample Kolmogorov-Smirnov normal test for male partners: p=0.2, normal parameters: mean = -8.4, SD = 0.63; one-sample Kolmogorov-Smirnov normal test for female partners: p=0.2, normal parameters: mean = 8.8, SD = 0.76.

Explanation of the decision situation to the participants

Below we give an account of the instructions that we gave to the participants of our experiment. As explained in the main text, the Trust Game was part of a larger experiment. We left out those parts of the instructions that refer to parts of the larger experiment that are not relevant to the Trust Game.

General instructions:

You will enter a series of interactions. Your decisions will be matched with the decisions of other participants in the experiment; every interaction will involve a new person. As a result of your decisions, you will earn points, which at the end of the experiment will be converted to real money. All your decisions will be treated anonymously! Other participants will not know your decisions at any step of the experiment.

There are various types of interaction, each corresponding to a different decision situation. Please try to imagine these situations as if they were happening in your real life, or try to remember how you have behaved in similar situations before. Please ask the experimenters for help if anything is unclear.

Introducing anonymous interactions:

During this part of the experiment, you will face a situation, where you will have to make a decision, which will be matched with the decision of another participant in the experiment. Both of you receive a payoff from the interaction, but neither of you knows who the partner is.

Introducing personalized interactions:

Now you are facing the same situation that you have just encountered, but this time you can first watch a silent video of your interaction partner. Only you get this additional piece of information – your interaction partner will not watch your video at the same moment. The outcome of the interaction will be determined by your own decision and the decision your partner (the person on the video) made in an anonymous situation.

Introducing the Trust Game:

The decision situation consists of two separate parts. In the first part, you have the role of a farmer, and in the second part, you have the role of a market seller. For each part, you will receive a payoff, which will depend on your own decision and the decision of your interaction partner in that part. Neither you nor your partner will be informed about the payoffs received; your payoffs will be added to those you accumulated in the earlier parts of the experiment.

The decision situation is as follows: A farmer living in a village has some goods for sale, which are worth 50 points when sold in the village. A market seller (unknown to the farmer) who is passing through the village suggests taking the farmer's products to the city market and selling them there for 150 points. The market seller states that she/he will come back in the evening and share the profit with you. The farmer has two options: to refuse the deal or to hand over the goods to the market seller. In the first case, the famer keeps 50 points, while the market seller receives 0 points. If the farmer hands over the goods for sale, it will be solely the decision of the market seller how many of the 150 points she/he returns to the farmer. In principle, the market seller can avoid paying back any points (thus keeping 150 points, while the farmer gets 0 points), by not returning at all to the village. The market seller can also decide to return 50, 75, or 100 of the 150 points to the farmer, keeping 100, 75, or 50 points for her- or himself, respectively. The farmer cannot enforce to receive a larger amount than offered by the market seller, as she/he does not know at what price the goods were actually sold on the city market. After the transaction, the market seller departs, and famer and market seller will presumably not meet again.

Decision to be made in the role of a farmer:

Now imagine that you are the farmer, who has some goods for sale. Your interaction partner (another participant of the experiment) takes on the role of a market seller. You have two options:

A: I refuse the deal and sell my goods in the village; my payoff will be 50 points.

B: I hand over my goods to the market seller; depending on the market seller's behaviour, I will receive a payoff of 0, 50, 75, or 100 points.

Please, make your choice!

Decision to be made in the role of a market seller:

Now imagine that you are a market seller and that a farmer has handed over goods to you that you have sold for 150 points on the city market. You have four options:

A: I return 100 points to the farmer and keep a payoff of 50 points for my work.

B: I return 75 points to the farmer and keep a payoff of 75 points for myself.

C: I return 50 points to the farmer and keep a payoff of 100 points for myself.

D: I do not return to the village and keep a payoff of 150 points.

Please, make your choice!
